# Surveillance of diphtheria in the Netherlands between 2000–2021: cutaneous diphtheria supersedes the respiratory form

**DOI:** 10.1186/s12879-023-08388-5

**Published:** 2023-06-21

**Authors:** Jelte Elsinga, Dimphey van Meijeren, Frans Reubsaet

**Affiliations:** 1 Centre for Infectious Disease Control, National Institute for Public Health and the Environment (RIVM), Antonie Van Leeuwenhoeklaan 9, 3721 MA Bilthoven, The Netherlands; 2 Department of Medical Microbiology and Infection Prevention, Amsterdam UMC, Meibergdreef 9, 1105 AZ Amsterdam, The Netherlands

**Keywords:** Diphtheria, Human-to-human transmission, Vaccination coverage, Laboratory surveillance, Epidemiology, Diphtheriae exotoxin

## Abstract

**Background:**

Diphtheria is a severe respiratory or cutaneous infectious disease, caused by exotoxin producing *Corynebacterium diphtheriae*, *C. ulcerans* and *C. pseudotuberculosis*. Diphtheria is once again prevalent due to breakdown of immunisation programmes, social disruption and unrest.

**Aim:**

This study describes the notified diphtheria cases in the Netherlands between 2000–2021 and isolates that were sent to the National Institute for Public Health and the Environment (RIVM).

**Methods:**

File investigation was performed including all notified cases and isolates of *C. diphtheriae*, *C. ulcerans* and *C. pseudotuberculosis* that were tested for toxin production using a toxin-PCR and Elek test. An exploratory review was performed to understand transmission in populations with a high vaccination uptake.

**Results:**

Eighteen diphtheria notifications were made with confirmed toxigenic *C. diphtheriae* (*n* = 9) or *ulcerans* (*n* = 9) between 2000 and 2021. Seventeen (94.4%) presented with a cutaneous infection. All cases with a suspected source abroad (*n* = 8) concerned infection with *C. diphtheriae*. In contrast, 9/10 cases infected in the Netherlands were caused by *C. ulcerans*, a zoonosis. Secondary transmission was not reported. Isolates of *C. ulcerans* sent to the RIVM produced more often the diphtheria exotoxin (11/31; 35%) than *C. diphtheriae* (7/89; 7.9%).

**Conclusion:**

Both human-to-human transmission of *C. diphtheriae* and animal-to-human transmission of *C. ulcerans* rarely occurs in the Netherlands. Cases mainly present with a cutaneous infection. Travel-related cases remain a risk for transmission to populations with low vaccination coverage, highlighting the importance of immunization and diphtheria control measures.

**Supplementary Information:**

The online version contains supplementary material available at 10.1186/s12879-023-08388-5.

## Introduction

Diphtheria is a respiratory or cutaneous infectious disease, caused by three different species of *Corynebacterium*: *C. diphtheriae*, *C. ulcerans* and *C. pseudotuberculosis,* which have the potential to produce a potent exotoxin when the toxin gene of the corynebacteriophage is present [1, 2]. *C. diphtheriae* and *C. ulcerans* are the most pathogenic in humans [3]. Respiratory diphtheria causes upper respiratory tract symptoms and is known by a pseudomembrane in the throat, while cutaneous diphtheria causes skin lesions. Both forms can be responsible for cases of systemic diphtheria that can result in, among others, myocarditis, neuritis and sudden cardiac death [1, 2]. *C. diphtheriae* is transmitted via droplets or via direct contact (kissing, contact with wound). Transmission of *C. ulcerans* mainly occurs through animals (cattle, horses, dogs, cats) or unpasteurised milk.

Diphtheria is a much-feared infectious disease that caused numerous outbreaks and deaths. A major epidemic of diphtheria in Europe took place in World War II, with approximately 1 million cases and a 15% death rate for respiratory diphtheria [3]. Later, from 1990 to 1997, more than 115,000 cases and 3000 deaths were documented in the Russian Federation [4]. Globally, diphtheria outbreaks remain a threat in countries or groups where vaccination coverage is low. Annually, an average of 8,243 diphtheria cases were reported to WHO between 2000 and 2021, with the majority (65.3%) being reported in South-East Asia (mainly from India, Nepal and Indonesia) [5]. Recent (between January 2016 and February 2019) major outbreaks of diphtheria were reported in the context of humanitarian crises: among Rohingya refugees in Bangladesh (8,403 patients), in Yemen (3,340 patients) and in Venezuela (2,512 patients) [3, 6]. In Europe, between 32–73 cases were reported annually from 2010 to 2021, of which 47% were identified as *C. diphtheriae* in 2017 and 2018 [7, 8].

The low numbers of diphtheria cases in the Netherlands are mainly attributed to the ongoing vaccination program that started in 1953. During the time of this study the immunization schedule consisted of a vaccination at three, five and eleven months, followed by boosters at the ages of four and nine [9]. The national vaccination coverage of the infant series for those born in 1970 – 2018 ranged from 88.7% to 95.8%. However, the so-called Bible Belt, which is a belt-shaped area in The Netherlands stretching from the southwest to the northeast where religious population groups are concentrated, has had a coverage of the infant series below 80% in the past decades [10]. It is assumed that group immunity for diphtheria arises with a vaccination coverage of more than 80–85% [1]. Interestingly, a seroprevalence study of diphtheria antibodies in the Dutch population (aged between 35–39 years, 26–30 year after their last vaccination) revealed that 5.4% had no measurable protection (< 0.01 IU/ml) against diphtheria. The proportion of persons with no protection was higher among older, not (completely) vaccinated, age groups and among orthodox protestants [11, 12].

Based on the public health act (WPG), toxigenic diphtheria is a notifiable disease in The Netherlands. When *C. diphtheriae* or *C. ulcerans* is diagnosed, toxin production is tested promptly by a diphtheria toxin PCR and Elek test and prevention or control measures will be started/continued when these tests are positive [1, 13]. Nasopharyngeal swabs from close contacts are cultured to identify transmission. Depending on the identified species and disease presentation (cutaneous or respiratory), disease control measures can include isolation of the case and offering (close) contacts information, antibiotic prophylaxis and a vaccination update. Species can be defined promptly and this might aid in decision making of infectious disease control, since human-to-human transmission of *C. ulcerans* has not been described in literature.

Between 2000–2021, human-to-human transmission of *C. diphtheriae* has only rarely been described in Europe [8]. This study analyses the transmission of diphtheria before the rise of diphtheria cases amongst migrants, starting in Europe in 2022 [14]. In order to gain insight into whether and how often (human-to-human) transmission occurred in The Netherlands (a population with a high national vaccination coverage, but including areas with coverages < 80%), and to help decision making in diphtheria control measures, our study aims were as follows:

- to describe the reported diphtheria cases from 2000 up to and including 2021 in The Netherlands
- to describe proportions of toxin production of different *Corynebacterium* subspecies
- to review existing literature on human-to-human transmission of diphtheria in populations with a high vaccination coverage.

## Methods

Two sources from the RIVM were investigated for this study: the laboratory surveillance and the files of notifications for infectious disease control. These sources differ, since the notifications for infectious disease control only include cases that strictly match case definitions (as are defined in national guidelines), whilst laboratory surveillance include all human and animal samples that were sent by laboratories to the RIVM for identification, without a strict case-definition. Most human strains in local medical laboratories identified as *Corynebacterium diphtheriae* or *C. ulcerans* (nowadays mostly using Matrix-assisted Laser Desorption Time of Flight Mass Spectrometry (MALDITOF MS)), are submitted to the RIVM for diphtheria toxin gene PCR and Elek test (notifiable case definition testing), since most local laboratories do not have a toxin PCR or Elek test. Laboratory surveillance therefore also includes human strains that tested toxin PCR- and/or Elek test negative. In addition, the laboratory surveillance includes animal strains (*Corynebacterium diphtheriae*, *C. ulcerans* or *C. pseudotuberculosis)* that are submitted voluntary.

### File investigation of notifications

Health professionals are required to report diphtheria cases to their local Public Health Service (GGD) within 24 h if a patient has symptoms corresponding to diphtheria and a proven toxigenic *C. diphtheriae* or, since 2009, *C. ulcerans*. The GGDs then report the case data to the RIVM, which in turn shapes the national diphtheria surveillance. The following data of the reported cases were collected: diagnosis date, age index, sex index, clinical manifestation, pathogen, vaccination status index, source (country/person/animal) pathogen, hospitalization, mortality, number of secondary cases, number of first-ring contacts, vaccination status/treatment/diagnosis of first-ring contacts. Data was collected and analyzed in Excel.

### Laboratory surveillance

For each strain (*n* = 150) the RIVM received for laboratory analyses regarding detection of toxin production between 2000–2021, the following information was collected: date of receipt, the species and biotype, the physical origin (i.e. a human or a non-human origin, sex and age for human strains, place on the body and/or type of wound), the probable country of origin, the toxin PCR test result and, if performed, the Elek test result.

The toxin PCR used in the surveillance was validated in an unpublished study [15]. A schematic presentation of the PCR-cycles is provided in Additional file 1. In case the toxin PCR was positive, the modified Elek test was performed in duplo according to Engler et al. [16], with positive control (PW8 ATCC 11952) and negative (ATCC 11951) control strains. The species identification and biotyping was confirmed by the phenotypical characteristics [17–22], partial 16S rRNA gene PCR and subsequent Sanger sequencing and MALDITOF MS (Bruker Nederland, Leiderdorp). The reliability of the MALDITOF MS identification was only investigated with a limited number of *C. ulcerans* (*n* = 8) and *C. pseudotuberculosis* (*n* = 4) strains, in contrast to *C. diphtheriae* (78 strains) [23]. Since the identity of the strains in the Bruker database was not clear for all entries, only the scores for the type strains of *C. ulcerans* and *C. pseudotuberculosis* were used.

The non-toxic species *C. rouxii* and *C. silvaticum* were excluded by comparing their 16S RNA gene sequences of with the sequences of the strains in this study [24]. *C. diphtheriae* subspecies *lausannense* was considered to be a synonym of biotype Belfanti, recently classified as species *C. belfantii* [25].

### Statistical analysis laboratory surveillance

Three non-Corynebacteria, multiple isolates from one patient (*n* = 3) and one strain identified as Mitis/Intermedius were excluded from analyses, resulting in 143 isolates for analysis. Weakly positive Elek test results were characterized as positive.

Statistical analysis took place in R version 4.1.0 and aimed at examining the association between the species or biotypes and both the toxin PCR test result and the Elek test result. The toxin PCR test result was the main outcome variable, since a positive toxin PCR is indicative for toxin production. A Fisher-Freeman-Halton exact test was performed, as the assumptions for the chi-squared test were violated. If an association was considered statistically significant (*p*-values ≤ 0.05), additional multiple comparisons were performed with *p*-value adjustments by the Bonferroni method.

### Explorative literature study

Special attention was paid to the question of whether the scientific literature describes diphtheria-transmission in a population with a high vaccination-uptake. Pubmed was searched for articles describing transmission in or by vaccinated persons. The Pubmed search strategy was as follows: *"diphtheria"[Title] AND ("outbreak"[Title/Abstract] OR "cluster"[Title/Abstract] OR "transmission"[Title/Abstract]) AND "vaccination"[Title/Abstract]*.

The search was performed on the 10^th^ of August 2021. No limits were set to publication date. One researcher reviewed all titles and abstracts, and if needed the full text to determine whether transmission of diphtheria involving vaccinated individuals was described. Literature in languages other than English, Dutch or Spanish were excluded.

## Results

### File investigation of notifications

Table 1 provides an overview of the diphtheria cases notified between 2000 and 2021, and their characteristics. It should be noted that *C. ulcerans* was notifiable from 2009 onwards. Between 2000—2010 no diphtheria was reported, but two (one in 2002 and 2007) human toxin PCR- and Elek-positive *C. ulcerans* were identified in the laboratory surveillance. In addition, all Elek tests were repeated with an improved modified test in 2022. The results presented here are the results of the repeated tests (performed in 2022). Two *C. ulcerans* samples that earlier tested negative in 2015 and 2018, had a positive Elek test in 2022. The latter tests were not notified and are therefore missing in Table 1. Four diagnoses were confirmed abroad or by a local laboratory, by which data on toxin PCR test was missing in three cases and data on Elek test in four cases. Since these strains were no longer available, we were unable to confirm by the laboratory surveillance at the RIVM whether these cases met the case definition. There was a difference in the records for two cases, that documented *C. diphtheriae* as the pathogen in the notification, while for these cases *C. ulcerans* was the reported pathogen in the laboratory surveillance. *C. ulcerans* was considered the correct pathogen here because we assumed the laboratory data to be more reliable for species determination.

**Table 1:** Diphtheria cases notified between 2000 and 2021, and their characteristics^a^

**Case number**	**Year of diagnosis**	**Age**	**Sex**	**Region**	**Toxin PCR test**	**Elek test**	**Clinical manifest-ation**^**b**^	**Pathogen**^**c**^	**Vaccination status**	**Most probable source (country)**	**Most probable source (animal)**	**Hospitali-zation**	**Mor-tality**	**Close contacts**	**Secon-dary cases**
1	2011	61–70	F	West	Pos	Pos	Cut	*C. diph*	> 3 doses	Gambia	n/a	N	N	-	0
2	2012	61–70	M	Nord	Pos	Pos	Cut	*C. diph*	> 3 doses	Gambia	n/a	Y	N	-	0
3	2014	31–40	F	West	Pos	Pos	Cut	*C. diph*	0 doses	Ethiopia	n/a	N	N	-	0
4	2015	51–60	M	South	Pos	Pos	Cut	*C. diph*	> 3 doses	Indonesia	n/a	N	N	-	0
5	2015	41–50	M	South	Pos	Pos	Cut	*C. ulc*	Vaccinated, doses unknown	The Netherlands	n/a	Y	N	-	0
6	2015	41–50	M	North	-	-	Cut	*C. ulc*	-	The Netherlands	-	N	N	-	0
7	2015	41–50	M	East	-	-	Cut	*C. diph*	-	The Netherlands	Cow	N	N	-	0
8	2016	51–60	F	West	Pos	Pos	Cut	*C. ulc*	> 3 doses	The Netherlands	Possibly dogs	N	N	-	0
9	2016	31–40	M	West	Pos	Pos	Cut	*C. diph*	> 3 doses	Indonesia	n/a	N	N	-	0
10	2016	61–70	M	North	Pos	Pos	Cut	*C. ulc*^d^	-	The Netherlands	Cat	N	N	-	0
11	2017	51–60	F	East	Pos	Pos	Resp	*C. ulc*	0 doses	The Netherlands	Dog	Y	N	-	0
12	2017	51–60	M	West	-	-	Cut	*C. diph*	> 3 doses	Sri Lanka	n/a	N	N	-	0
13	2017	21–30	F	West	Pos	Pos	Cut	*C. diph*	> 3 doses	Thailand / Indonesia	n/a	N	N		-
14	2018	51–60	M	North	Pos	Pos	Cut	*C. ulc*	Vaccinated, doses unknown	The Netherlands	Possibly dogs and/or cats	N	N	4	0
15	2018	51–60	M	West	Pos	Pos	Cut	*C. ulc* ^d^	3 doses	The Netherlands	Possibly a dog	N	N	-	0
16	2020	81–90	M	East	Pos	Neg	Cut	*C. ulc*	-	The Netherlands	Possibly dog and/or donkeys	Y	N	3	0
17	2020	21–30	F	North	Pos	-	Cut	*C. ulc*	> 3 doses	The Netherlands	Possibly cat and/or dog	Y	N	1	0
18	2020	31–40	F	West	Pos	Pos	Cut	*C. diph*	0 doses	Slovakia	n/a	Y	N	6	0

*Legend*: Meaning abbreviations: - missing value, *n/a* not applicable, *Y* Yes, *N* No, *F* Female, *M* Male

^a^*C. ulcerans* was notifiable from 2009 onwards

^b^Resp = respiratory; Cut = cutaneous

^c^*C. diph* = *C. diphtheriae*; *C. ulc* = *C. ulcerans*

^d^Discrepancy between the identification in the records of notifiable cases (*C. diphtheriae)* and laboratory surveillance (*C. ulcerans)*. We considered the laboratory surveillance as the correct

### Transmission

If transmission in The Netherlands was suspected (*n* = 10, 56%), almost all cases (*n* = 9, 90%) involved an infection with *C. ulcerans*. In these cases, dogs, a cat or a donkey were documented as possible source. These possible sources were often not confirmed because testing was not performed or the test results were not communicated. In one *C. diphtheriae* case, infected in The Netherlands, a cow was documented as possible source. Because this pathogen was diagnosed outside of the RIVM, the laboratorial records were not available. All other *C. diphtheriae* cases (*n* = 8) were infected abroad. There were no *C. ulcerans* cases documented with a possible source abroad.

### Clinical data

One case presented with respiratory diphtheria and was admitted to the hospital. In total 6 cases (33%) were admitted to the hospital, of which four infected by *C. ulcerans* and two by *C. diphtheriae*. Most cases (*n* = 11, 61%) were vaccinated with at least one dose. No patients died.

### Contacts and prophylaxis

Information about contacts was available for four of the cases. Between one and six close contacts were reported. These were treated with a (booster) vaccine, if the immune status for diphtheria was judged insufficient. No antibiotics were given. It was not documented whether nasopharyngeal swabs from close contacts were cultured, however, no secondary cases were reported.

### Laboratory surveillance

Out of the 143 samples, 120 were human and 23 non-human. Table 2 provides an overview of the toxin PCR test results per species and biotype of both the human and non-human strains. The results show that *C. diphtheriae* Belfanti did not test toxin-positive. Also *C. pseudotuberculosis* Ovis did not test toxin-PCR positive in our data. The highest percentage of toxin PCR-positive results (42%) was seen in *C. ulcerans.*

**Table 2:** Human and non-human diphtheria strains admitted to the RIVM between 2000 and 2021, by species and biotype, and their toxin PCR test results

***Species and biotype***	**All samples (** ***n*** ** = 143)**		**Human samples (** ***n*** ** = 120)**	
	**Toxin PCR-positive (*n* = 22)**^**1**^	**Toxin PCR-negative (** ***n*** ** = 121)**	**Toxin PCR-positive (*n* = 18)**^**2**^	**Toxin PCR-negative (** ***n*** ** = 102)**
	**n (%)**	**n (%)**	**n (%)**	**n (%)**
*C. diphtheriae* Belfanti	0 (0)^1^	23 (100)	0 (0)^2^	23 (100)
*C. diphtheriae* Gravis	1 (4)^1^	25 (96)	1 (4)^2^	25 (96)
*C. diphtheriae* Intermedius	3 (27)	8 (73)	3 (27)	8 (73)
*C. diphtheriae* Mitis	3 (10)	26 (90)	3 (10)	26 (90)
*C. ulcerans*	15 (42)^1^	21 (58)	11 (35)^2^	20 (65)
*C. pseudotuberculosis* Ovis	0 (0)^1^	18 (100)	-	-

^1^Fisher-Freeman-Halton exact test showed the species and biotypes had significant different toxin PCR test results (*p*-value < 0.001). A higher toxin PCR-positive percentage for *C. ulcerans* compared with *C. diphtheriae* Belfanti (*p*-value = 0.002)*, C. diphtheriae* Gravis (*p*-value = 0.01) *and C. pseudotuberculosis* Ovis (*p*-value = 0.01) was observed

^2^Fisher-Freeman-Halton exact test showed the species and biotypes had significant different toxin PCR test results (*p*-value = 0.001). A higher toxin PCR-positive percentage for *C. ulcerans* compared with *C. diphtheriae* Belfanti (*p*-value = 0.01) and *C. diphtheriae* Gravis (*p*-value = 0.04) was observed

All *C. pseudotuberculosis* Ovis (*n* = 18) and five *C. ulcerans* samples from an animal were excluded to perform the statistical analysis on the human strains only. Again we observed the highest percentage (35%) of PCR positive result in *C. ulcerans.* Table 2 provides an overview of the toxin PCR test results per species and biotype of the human strains.

A Fisher’s exact test was performed on all samples to test whether the species and biotypes had significant different toxin PCR test results (*p*-value < 0.001). In addition, pairwise comparisons showed a higher toxin PCR-positive percentage for *C. ulcerans* compared with *C. diphtheriae* Belfanti (*p*-value = 0.002)*, C. diphtheriae* Gravis (*p*-value = 0.01) *and C. pseudotuberculosis* Ovis (*p*-value = 0.01).

Performing the same analyses only on the human samples showed that *C. ulcerans* tested more often toxin PCR positive than *C. diphtheriae* Belfanti (*p*-value = 0.01) and *C. diphtheriae* Gravis (*p*-value = 0.04)*.*

In only one strain, a *C. ulcerans,* we found a negative Elek test result after a positive toxin PCR test result (Table 1).

### Literature study

The search term used for the literature study yielded 89 search results, of which six articles described human-to-human transmission involving vaccinated individuals. These are described in Table 3.

**Table 3:** Summary of articles describing transmission of diphtheria in a vaccinated population

Authors, title of manuscript	Article type	Summary of relevant information
Edwards et al. [26] *Transmission of toxigenic Corynebacterium diphtheriae by a fully immunised resident returning from a visit to West Africa, United Kingdom, 2017*	Case report	In 2017, for the first time in 30 years, transmission of diphtheria within the UK was confirmed. A cutaneous infection with a toxigenic (Elek +) *C. diphtheriae* was diagnosed in a returning traveller (teenager) who had been adequately vaccinated. There were nine close contacts, of which one 81-year-old (vaccination status unknown) developed a mild symptomatic diphtheria. The three close contacts of the 81-year-old contact tested negative for toxigenic *C. diphtheriae.*
Truelove et al. 2020 [27] *Clinical and Epidemiological Aspects of Diphtheria: A Systematic Review and Pooled Analysis*	Systematic review with pooled analyses *(the other articles in this table were not cited by this review)*	From a systematic literature search with pooled analysis, nine different questions were analysed. Between March–May 2018, 6934 articles were found, of which 266 were included in the study. The incubation period of diphtheria was found to have an IQR of 0.8–3.4 days. 88% of fully vaccinated infected people became asymptomatic carriers. Symptomatic and asymptomatic carriers of *C. diphtheriae* and *C. ulcerans* carry the bacteria for an average of 18.5 days, and 5% carry the bacteria for more than 48 days. Antibiotic treatment shortens colonisation of the bacteria to an average of 5.2 days. The case fatality rate was found to be 15.8% in unvaccinated persons and 1.0% in vaccinated persons. As humans are the only carriers of *C. diphtheriae*, the researchers conclude that cutaneous diphtheria plays an important role as a reservoir in the inter-epidemic periods.
Jané et al. [28] *A case of respiratory toxigenic diphtheria: contact tracing results and considerations following a 30-year disease-free interval, Catalonia, Spain, 2015*	Case report	In Catalonia, an unvaccinated child with no travel history was infected with a toxigenic (Elek +) *C. diphtheriae*. A ring investigation showed that nine of the 178 contacts identified had a positive throat swab for a toxigenic *C. diphtheriae*. These nine asymptomatic carriers had all been vaccinated and had 39 family members of whom one tested positive. The researcher mentions that in the context of transmission, ring testing and treatment of carriers appears to be important. Vaccinated people seemed to be less infectious in this study.
Alberto et al. [29] *Cutaneous ulcers revealing diphtheria: A re-emerging disease imported from Indian Ocean countries?*	Several concise case reports	Thirteen cases of cutaneous diphtheria in France and La Réunion between 2015 and 2018 were described. All were most likely infected in a country bordering the Indian Ocean. Of the thirteen cases, all were caused by *C. diphtheriae* of which five were toxigenic (Elek +). One contact developed cutaneous lesions (non-toxigenic *C. diphtheriae*).
Ohuabunwo et al. [30] *Respiratory diphtheria among highly vaccinated military trainees in Latvia: improved protection from DT compared with Td booster vaccination*	Outbreak report	Transmission of a toxigenic *C. diphtheriae* occurred at a military training camp in Latvia in 2000. There was a high level of close contact, with drinking cups being shared. Of the 207 people present, more than 85% had been vaccinated against diphtheria with five or more doses. There were 45 (22%) symptomatic cases and 79 (38%) carriers of the toxigenic *C. diphtheriae*. The course of disease was mild, except for one person who developed severe myocarditis. An outbreak of mild diphtheria among vaccinated persons can occur during intense exposure.
Rappuoli et al. [31] *Molecular epidemiology of the 1984–1986 outbreak of diphtheria in Sweden*	Molecular outbreak investigation	There were two diphtheria outbreaks, in Göteborg and Stockholm in Sweden from 1984–1986, which occurred mainly among alcoholics and drug users. 17 cases of clinical diphtheria had mainly (16/17) low antitoxin titres (< 0.01 IU/mL) and 65 carriers were identified, 92% of which had higher antitoxin titres (> 0.16 IU/mL). Based on analysis of strains of *C. diphtheriae* among alcoholics and drug addicts (and their contacts), the researchers concluded that vaccinated asymptomatic carriers most likely played a role as a reservoir for *C. diphtheriae.*

## Discussion

Between 2000 and 2021, diphtheria cases were only reported rarely in The Netherlands and no secondary human transmission was reported. Cutaneous infections were predominant (17/18) compared with the respiratory form. Almost all cases (9/10) that were infected in The Netherlands were infected by the species *C. ulcerans.* An infection with *C. diphtheriae* was most often contracted abroad, in African or South-East Asian countries.

From 2014 to 2018, European countries reported a total of 254 (38–65 annually) cases of toxigenic *C. diphtheriae* or *ulcerans* [16]. Most European cases were reported by France and Germany. The low incidence of diphtheria in The Netherlands and Europe is likely explained by the high vaccination coverage. In addition, *C. ulcerans*, which accounts for most autochthonous cases in The Netherlands, has in literature not been described to cause outbreaks via human-to-human transmission. The high vaccination coverage and current diphtheria control policies largely protect populations in The Netherlands with a lower vaccination coverage than the threshold for group immunity (< 80%) [1]. Nevertheless, the current increase (fall 2022) in diphtheria among immigrants, mainly from countries with poor immunisation programs, in Europe and The Netherlands, does put these populations at risk [14].

All vaccinated diphtheria cases notified between 2000 and 2021 were aged ≥ 20 years, indicating that the high uptake of vaccines and the immunization schedule of the Netherlands adequately protects children, but that waning immunity might make people susceptible for diphtheria again over time. Unfortunately, additional parameters of cases infected after complete vaccination were not available and we were therefore unable to explore immunological explanations for these infections.

Little is known about *C. ulcerans* carrier incidences in Europe, making the occurrence and conditions of animal-to-human transmission difficult to estimate. In addition, *C. ulcerans* has only been compulsorily notifiable in The Netherlands since 2009. Therefore, surveillance data on *C. ulcerans* might be incomplete since it is unclear if, and to what extent, laboratories actually submit all strains of *C. ulcerans* for toxin PCR and Elek testing for toxigenicity.

Since 2014, in The Netherlands, Belgium [32] and the United Kingdom [33], more toxigenic Corynebacteria have been diagnosed in cutaneous infections than before 2014. This increase in cutaneous infections can be explained by new developments in diagnostics, such as the use of MALDI-TOF MS. This suggests that more sensitive diagnostics lead to completer (and quicker) treatment of cutaneous diphtheria, which is positive in the light of infectious disease control, since cutaneous diphtheria can play an important role as a reservoir during inter-epidemic periods [27]. In our surveillance, 7.9% of the analysed samples of *C. diphtheriae* were toxigenic. *C. ulcerans* was the species with the highest percentage of toxigenic isolates (42% in all samples and 35% in human samples), which showed to be significantly higher compared with *C. diphtheriae* Belfanti, *C. diphtheriae* Gravis and *C. pseudotuberculosis* Ovis. Isolates of *C. diphtheriae* Mitis and Intermedius were toxin producing in 10.3% and 27.3% of the isolates, respectively. Research from the United Kingdom and Spain National Reference Laboratories [33, 34], showed similar results between 2009 and 2019, concerning percentages of toxigenic *C. ulcerans* and *C. diphtheriae*. *C. diphtheriae* Mitis showed the highest percentage of toxigenic isolates, while the isolates of biotypes Belfanti and Gravis were not toxigenic [34]. Older studies between 1985–2003 showed higher percentages of toxigenic isolates in the United Kingdom (85% (69/81) of *C. ulcerans*) [35], Algaria (59% *C. diphtheriae* Mitis (72/122) and 28% *C. diphtheriae* Gravis (2/7)) [36], Russia and Ukraine (74% *C. diphtheriae* Mitis (14/19), 94% *C. diphtheriae* Gravis (50/53)) [37]. These differences might be caused by different diagnostic methods and case definition, that hindered detection of non-toxigenic species.

Comparing our results with recent studies in similar contexts [33, 34] and with older studies from a different context [36, 37] illustrates that surveillance of *Corynebacterium* causing diphtheria remains highly dependent on surveillance infrastructure and diagnostic possibilities. It is likely that nowadays in the European countries, MALDI-TOF MS detects more *Corynebacterium* probably causing diphtheria than in earlier times. This increased detection may have led to prevention of transmission.

Relatively few outbreaks of *C. diphtheriae* are described in the literature among fully vaccinated persons. However, a diphtheria outbreak in Latvia among military personnel shows that transmission can occur even among fully vaccinated persons if there is frequent and close contact [30]. Although several studies describe transmission through fully (and recently) vaccinated persons [26–30], it seems that vaccinated persons develop fewer symptoms when infected [27, 30], reducing the risk of transmission by about 60% compared to unvaccinated persons [23]. Vaccinated and unvaccinated people carry *C. diphtheriae* for a shorter average period (5.2 vs. 18.5 days) when receiving antibiotic treatment [27].

Considering the above, the current policy in the Netherlands—antibiotic treatment of first contacts regardless of vaccination status [1]—is a well-considered choice. As vaccinated persons are still colonised for an average of 5.2 days even after antibiotic treatment, it could be considered to broaden the current exclusion period from school and work (up to 2 days after starting antibiotic therapy [1]). In addition, vaccinated persons with cutaneous diphtheria may act as a reservoir for *C. diphtheriae*, possibly introducing it into a low-vaccinated population—resulting in a diphtheria outbreak [31]. However, given that current policy has only led to eighteen notified diphtheria cases, and no secondary cases in the Netherlands in the last ten years (up to and including 2021), the current guidelines, and the high vaccination coverage, seem to adequately control diphtheria transmission. Nevertheless, the aforementioned literature, and the (potential) increase of diphtheria cases in Europe, whether or not among immigrants, gives no reason to loosen the current Dutch guidelines for diphtheria.

Limitations of this study correspond to those of medical record research. Furthermore, the national surveillance is dependent on samples and notifications from clinics and GGDs. Although diphtheria surveillance is a structured process in The Netherlands, we discovered missing data or differences between laboratory data and data from the responsible GGD, which highlight vulnerabilities within the surveillance system. Follow-up research can focus on diphtheria transmission in countries with a similar vaccination programme and uptake to The Netherlands. In addition, the role of (domestic) animals in the transmission of *C. ulcerans* could be further investigated, as part of a One Health approach.

Based on this study and the reviewed literature, we conclude that the risk of human-to-human transmission of *C. diphtheriae* is minimal in the Netherlands, a country with a high vaccination coverage. Transmission via (domestic) animals leads in rare cases to a cutaneous diphtheria caused by *C. ulcerans*. *C. ulcerans*, when detected, did produce most often the diphtheria exotoxin. Cases with *C. diphtheriae* were mostly infected abroad. Diphtheria prevention and control in the Netherlands remains important for public health, especially when considering the existence of populations with a low vaccination coverage and the threat of travel related cases.

## Supplementary Information

**Additional file 1.**

## Data Availability

The datasets used and/or analysed during the current study are available from the Dimphey van Meijeren (dimphey.van.meijeren@rivm.nl) on reasonable request.
