# Supplementary material for: Surveillance of diphtheria in the Netherlands between 2000–2021: cutaneous diphtheria supersedes the respiratory form
Source: BMC Infect Dis. 2023 Jun 21;23:420. doi: 10.1186/s12879-023-08388-5 (PMC10283224; doi:10.1186/s12879-023-08388-5)
Supplement: Supplementary file 1 — Additional file 1. [file 12879_2023_8388_MOESM1_ESM.docx]

|  | Temperature (°C) | Time (minutes) | Number of cycles |
| --- | --- | --- | --- |
| Pre-heat | 95 | 10 | 1 |
| Denaturation | 95 | 0.5 |  |
| Annealing | 66 | 0.5 | 2 |
| Extention | 72 | 1 |  |
| Denaturation | 95 | 0.5 |  |
| Annealing | 64 | 0.5 | 2 |
| Extention | 72 | 1 |  |
| Denaturation | 95 | 0.5 |  |
| Annealing | 62 | 0.5 | 2 |
| Extention | 72 | 1 |  |
| Denaturation | 95 | 0.5 |  |
| Annealing | 60 | 0.5 | 2 |
| Extention | 72 | 1 |  |
| Denaturation | 95 | 0.5 |  |
| Annealing | 58 | 0.5 | 2 |
| Extention | 72 | 1 |  |
| Denaturation | 95 | 0.5 |  |
| Annealing | 57 | 0.5 | 2 |
| Extention | 72 | 1 |  |
| Denaturation | 95 | 0.5 |  |
| Annealing | 56 | 0.5 | 2 |
| Extention | 72 | 1 |  |
| Denaturation | 95 | 0.5 |  |
| Annealing | 56 | 0.5 | 25 |
| Extention | 72 | 1 |  |
| Final extention | 72 | 7 | 1 |

**Additional file 1 - Table. Diphtheria toxin PCR scheme**

Legend: The toxin PCR was performed with forward primer CDTF591 (5' - CGC GTG TAG TGC TCA GC – 3'), and reverse-primer CDTR954 (5' - CAG GAT GCT CTA ATG CCG – 3'). Numbers correspond with the sequence described by Ratti et al. [1], resulting in a 364-nucleotides-sized-amplicon across the A and B part of the toxin sequence. 16S rRNA gene primers 16S8F (5’ - AGA GTT TGA TCM TGG YTC AG - 3’) and 16S575R (5’ - CTT TAC GCC CAR TRA WTC CG -3’) were included as internal control, resulting in a circa 568 nucleotides sized amplicon.

**Literature**
1. Ratti R, Rappuoli R, Giannini G. The complete nucleotide sequence of the gene coding for diphtheria toxin in the corynephage omega (tox+) genome. Nuclei Acids Research. 1983 Oct;11(19):6589-95.
